# Transient elastography for predicting esophageal/gastric varices in children with biliary atresia

**DOI:** 10.1186/1471-230X-11-41

**Published:** 2011-04-18

**Authors:** Voranush Chongsrisawat, Paisarn Vejapipat, Nipaporn Siripon, Yong Poovorawan

**Affiliations:** 1 Department of Pediatrics, Faculty of Medicine, Chulalongkorn University, Bangkok, Thailand; 2 Department of Surgery, Faculty of Medicine, Chulalongkorn University, Bangkok, Thailand

**Keywords:** Transient elastography, esophageal varices, gastric varices, biliary atresia

## Abstract

**Background:**

Transient elastography (TE) is an innovative, noninvasive technique to assess liver fibrosis by measuring liver stiffness in patients with chronic liver diseases. The purpose of this study has been to explore the accuracy of TE and clinical parameters in predicting the presence of esophageal/gastric varices in children with biliary atresia (BA) following portoenterostomy.

**Methods:**

Patients with BA status post portoenterostomy and normal children were recruited. Splenomegaly and presence of EV/GV were determined by physical examination and endoscopy, respectively. Aspartate transaminase to platelet ratio index (APRI) was used as a serum fibrosis marker. TE was performed by using FibroScan. Data was expressed as mean ± SD.

**Results:**

Seventy-three BA patients (male:female = 32:41; age 9.11 ± 5.64 years) and 50 normal controls (male:female = 19:31; age 11.00 ± 3.31 years) were enrolled. The liver stiffness score of BA patients was significantly higher than that of normal controls (27.37 ± 22.48 and 4.69 ± 1.03 kPa; *p *< 0.001). Patients with EV/GV had significantly higher liver stiffness score and APRI than those without EV/GV. As for EV/GV diagnosis, the areas under the receiver operating characteristic curve were 0.89 (95% CI 0.80 to 0.98) for TE and 0.87 (95% CI 0.78 to 0.96) for APRI, respectively. The sensitivity (and specificity) of TE (using a cut-off value of 12.7 kPa) and APRI (using a cut-off value of 1.92) in predicting EV/GV were 84% (77%) and 84% (83%), respectively, whereas the sensitivity (and specificity) of splenomegaly in predicting EV/GV were 92% (85%).

**Conclusions:**

Transient elastography is a useful tool for predicting the presence of EV/GV. In addition, basic physical examination, routine biochemical and hematological tests, are still worthwhile and correlate well with the presence of EV/GV in patients with BA post portoenterostomy.

## Background

Biliary atresia (BA) is a progressive, idiopathic, necroinflammatory process resulting in obliteration of the extrahepatic biliary tree. It is the most common cause of neonatal jaundice for which surgery is indicated and also the most common indication for liver transplantation in children. The pathogenesis of BA has remained a mystery. Most of the causal theories include defects resulting from a viral infection or toxin exposure, defects in morphogenesis, genetic predisposition, defects in prenatal circulation, and immune dysregulation [1]. The obstruction of bile flow results in worsening cholestasis, liver fibrosis, and cirrhosis, which lead to portal hypertension and a decline in hepatic synthetic function. Successful restoration of bile flow by portoenterostomy can be accomplished in 60 to 90% of patients, although 75% of them will progress to cirrhosis and eventually end-stage liver disease requiring liver transplantation [2]. Progression of liver disease following portoenterostomy is thought to be partly related to repeated attacks of cholangitis.

Liver fibrosis leads to development of portal hypertension and subsequent complications including ruptured esophageal and/or gastric varices (EV/GV), splenomegaly, and ascites. Bleeding from ruptured EV/GV can be fatal. Consequently, monitoring of the presence of EV/GV is useful for the commencement of prophylactic treatment. The standard diagnostic screening tool for EV/GV is endoscopy which is considered an invasive procedure in children.

The current gold standard for diagnosing liver fibrosis and cirrhosis is histological analysis of tissue obtained through liver biopsy. Nonetheless, even with sonography guidance, it still causes major complications such as bleeding requiring surgery, bile leak, visceral perforation or arteriobiliary fistula [3-6]. Furthermore it has some limitations including sampling errors along with intra-observer and inter-observer variation [7,8]. In recent years, efforts have been dedicated to the development of non-invasive tests to replace liver biopsy for fibrosis assessment and follow-up. Those tests include imaging, serum marker panels such as Fibrotest and aspartate transaminase to platelet ratio index (APRI), and last but not least, transient elastography [9-11].

Transient elastography (FibroScan) is an innovative, noninvasive, rapid, and reproducible technique to assess liver fibrosis by measuring liver stiffness in both adult and pediatric patients [12,13]. The principle of the apparatus is employment of pulse-echo ultrasound acquisition to track the propagation of the shear wave and to measure its velocity. The relation between the velocity and the tissue stiffness is that the stiffer the tissue, the faster the shear wave propagates [14]. Transient elastography has low intra- and inter-observer variability [15]. However, the data on liver stiffness measurement in children with chronic liver diseases is still limited.

The purpose of this study has been to evaluate the accuracy of transient elastography and clinical parameters in predicting the presence of EV/GV in children with BA following portoenterostomy.

## Methods

Children with BA who had undergone Kasai portoenterostomy at King Chulalongkorn Memorial Hospital were enrolled. A palpable spleen below the costal margin on physical examination (supine palpation) was considered splenomegaly. Presence of EV/GV was demonstrated by fiberoptic upper gastrointestinal endoscopic examination. Aspartate transaminase to platelet ratio index (APRI) was calculated as follows: AST (×40) × 100/platelet count (10^9^/L) [11].

Transient elastography was performed by a well-trained nurse using FibroScan 502 (Echosens, Paris, France). The measurements were performed by placing an ultrasound transducer probe of FibroScan on the intercostal space at the area of the right liver lobe with patients lying in a supine position with maximal abduction of the right arm. Probes with an external diameter of 5 mm and 9 mm were used in subjects with thoracic perimeters of ≤ 75 cm and 45-110 cm, respectively. The measurements were carried out until 10 validated results had been obtained with a success rate of at least 90%. The median value of 10 validated scores was considered the elastic modulus of the liver, and it was expressed in kilopascals (kPa). All subjects undergoing liver stiffness measurements had to be free from acute febrile illness.

This study was approved by the Ethics Committee, Faculty of Medicine, Chulalongkorn University. Written informed consent was obtained from the patients' guardians.

Continuous data were expressed as mean ± SD. Student's *t*-test and Chi square test were used to evaluate statistical significance of differences between groups. Receiver operating characteristic (ROC) curves were used to determine the reliability of the liver stiffness score and APRI for predicting the presence of EV/GV in postoperative patients with BA. Statistical analyses were performed using SPSS version 17.0 software (SPSS Inc, Chicago, IL). A *p *value below 0.05 was considered statistically significant.

## Results

Seventy-three BA patients (male:female = 32:41; age 9.11 ± 5.64 years) and 50 normal controls (male:female = 19:31; age 11.00 ± 3.31 years) were enrolled. BMI of BA patients was significantly lower than that of controls (17.69 ± 3.18 and 19.19 ± 3.79 kg/m^2^; *p *= 0.03). The liver stiffness score of BA patients was significantly higher than that of normal controls (27.37 ± 22.48 and 4.69 ± 1.03 kPa; *p *< 0.001). There were 39 patients with splenomegaly and 39 with EV/GV. Patients with EV/GV had significantly higher liver stiffness score and APRI than those without EV/GV (Table 1). Patients with splenomegaly also had significantly higher liver stiffness score than those without splenomegaly (38.88 ± 21.95 and 9.91 ± 5.95 kPa; *p *< 0.001).

**Table 1:** Comparison of liver stiffness score, aspartate transaminase to platelet ratio index (APRI), presence of splenomegaly, and platelet count between patients with and without esophageal and/or gastric varices

	Patients with esophageal and/or gastric varices	Patients without esophageal and/or gastric varices	*P *value
Liver stiffness score (kPa)	37.72 ± 21.55	10.97 ± 8.71	<0.001

APRI	6.59 ± 4.73	1.41 ± 1.25	<0.001

Presence of splenomegaly (no.)	36	3	<0.001

Platelet count (×10^3^/μL)	119.92 ± 85.23	220.74 ± 90.43	<0.001

**Figure 1:**
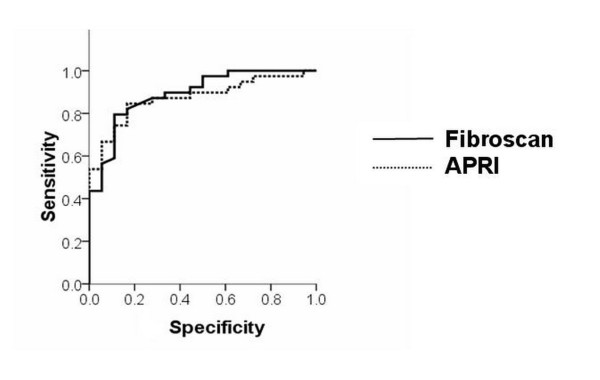
**ROC curve of FibroScan and aspartate transaminase to platelet ratio index (APRI) for diagnosis of esophageal and/or gastric varices**.

The diagnostic performance of Fibroscan, APRI, and the presence of splenomegaly for detection of EV/GV was shown in Table 2. As for EV/GV diagnosis, the areas under the receiver operating characteristic curve were 0.89 (95% CI 0.80 to 0.98) for FibroScan and 0.87 (95% CI 0.78 to 0.96) for APRI, respectively (Figure 1). The sensitivity (and specificity) of FibroScan (using a cut-off value of 12.7 kPa) and APRI (using a cut-off value of 1.92) in predicting EV/GV were 84% (77%) and 84% (83%), respectively whereas the sensitivity (and specificity) of splenomegaly in predicting EV/GV were 92% (85%).

**Table 2:** Performance of non-invasive markers for prediction of esophageal/gastric varices

	Splenomegaly	APRI	Fibroscan
Cut-off	-	1.92	12.7

Sensitivity (%)	92	84	84

Specificity (%)	85	83	77

PPV (%)	88	85	81

NPV (%)	91	83	81

Accuracy (%)	89	84	83

LR+	6.15	5.08	4.23

LR-	0.09	0.18	0.19

AUC	-	0.87	0.89

95% CI	0.85-0.99	0.78-0.96	0.80-0.98

APRI, AST-to-platelet ratio index; PPV, positive predictive value; NPV, negative predictive value; LR+, likelihood ratio for positive results; LR-, likelihood ratio for negative results; AUC, area under the curve; CI, confidence interval.

## Discussion

Patients with biliary atresia even after successful portoenterostomy may suffer from complications such as ruptured EV/GV which can be life threatening. A recent study has revealed that more than 50% of children with biliary atresia below the age of 2 years developed esophageal varices [16]. In addition, ectopic varices and portal hypertensive vasculopathy (eg, portal hypertensive gastropathy, enteropathy, and colonopathy) can also cause bleeding in patients with cirrhosis and portal hypertension.

Transient elastography is a reproducible test both in adults and children. Normal liver stiffness in normal adults is reported to be in the range of 4-6 kPa which is similar to that of normal children in this study [15]. Kazemi *et al *have shown that in adult patients with cirrhosis, the optimal cut-off value of the liver stiffness score for the diagnosis of esophageal varices is 13.9 kPa [17]. Furthermore, liver stiffness score of ≥13.6 kPa in adults with HCV-related cirrhosis is related to the presence of EV/GV which is also comparable to the figure of 12.7 kPa in the present study [18]. de Lédinghen V *et al *have evaluated liver stiffness measurement in children with various chronic liver diseases and found that transient elastography was correlated with the presence of esophageal varices and Metavir fibrosis score [12]. Chang *et al *also demonstrated that liver stiffness scores correlated well with the presence of esophageal or gastric varices in children with biliary atresia following portoenterostomy [19]. Increasing evidence has indicated that liver stiffness in children is related to liver fibrosis and its measurement is feasible. Liver stiffness measurement may represent a noninvasive alternative diagnostic tool to detect complications of portal hypertension in children with cirrhosis.

Screening for the presence of EV/GV by endoscopy is useful for considering prophylactic therapy to prevent bleeding, such as β-blockers to reduce portal pressure. Current guidelines recommend screening of all cirrhotic patients by endoscopy to estimate the risk of bleeding [20]. Unfortunately, thrombocytopenia is a common and challenging clinical disorder in patients with portal hypertension. It can increase the risk of bleeding associated with invasive procedures, for instance endoscopy. Standard therapy for thrombocytopenia typically consists of platelet transfusions, which may cause transfusion-related complications including viral or bacterial infection, alloimmunization, and febrile non-hemolytic reactions, which may occur in up to 30% of patients undergoing platelet transfusions [21]. This study has demonstrated that transient elastography is a helpful tool for predicting the presence of EV/GV. A recent report has shown that a high liver stiffness score in cirrhotic patients is significantly associated with portal hypertensive enteropathy besides EV/GV [22]. The substitution of transient elastography for endoscopy in children has several benefits in terms of avoiding the cost and complications of transfusion as well as the risk of general anesthesia or sedation during undergoing endoscopy. However, further large-scale studies are required to determine a liver stiffness cut-off value using transient elastography for best prediction of varices formation.

Transient elastography has several limitations. Obesity can also cause difficulties in measuring liver stiffness [23]. In the present study, five children in the control group were overweight (BMI > 25 kg/m^2^), but liver stiffness values were obtained without difficulty. Recently, it has been demonstrated that liver stiffness measurement is erratic for detecting fibrosis in patients with acute hepatitis [24,25]. The reasons underlying the high stiffness in acute liver damage are supposed to be related to hepatocyte swelling, cholestasis, or inflammatory cell infiltration in the acutely inflamed liver. In this study, this error was negligible. No subjects had acute febrile illness and thus, should not have acute cholangitis or viral infection which might cause acute liver damage.

The present study has also demonstrated that physical examination and hematological tests were well correlated with the presence of EV/GV. Fagundes *et al *observed that splenomegaly was useful as a screening test to predict the presence of EV [26]. Although the presence of splenomegaly may depend on the examiner's individual decision, our data have reinforced the importance of physical examination in children. A recent study demonstrated that APRI correctly diagnosed the presence of EV in 66% of adult cirrhotic patients [27]. A study in children with liver diseases revealed that at a cut-off score of 1.5, APRI detected the presence of EV in 67% of patients compared to 87% in the present study (data not shown) [28]. A recent large-scale study on adult cirrhotic patients revealed that a combination of non-invasive markers (Lok index and Forns' index) yielded an excellent negative predictive value (NPV) of over 90% to exclude clinically significant esophageal varice [29]. Our data showed that the presence of splenomegaly, APRI, and transient elastography also obtained a high NPV (80-90%) to exclude the presence of EV/GV in children with BA. These data emphasize the benefit of noninvasive tests in avoiding endoscopy in low risk cirrhotic patients.

## Conclusions

Transient elastography might be a new noninvasive, inexpensive method for detecting the presence of EV/GV in children with BA. In addition, basic physical examination, routine biochemical and hematological tests, are still worthwhile and correlate well with the presence of EV/GV in patients with BA post portoenterostomy.

## List of abbreviations used

APRI: aspartate transaminase to platelet ratio index; BA: biliary atresia; BMI: body mass index; EV/GV: esophageal and/or gastric varices; kPa: kilopascal; ROC: receiver operating characteristic; SD: standard deviation; TE: transient elastography

## Competing interests

The authors declare that they have no competing interests.

## Authors' contributions

VC, PV, NS and YP were involved in the conception and design of the study. VC participated in ethics approval, patient recruitment, performed the statistical and data analysis, proposal and manuscript preparation, and proposal writing. PV participated in patient recruitment, proposal and manuscript preparation. NS participated in patient recruitment and performed transient elastography. YP applied for funding, participated in patient recruitment, proposal and manuscript preparation, and drafting the manuscript. All authors read and approved the final manuscript.

## Pre-publication history

The pre-publication history for this paper can be accessed here:

http://www.biomedcentral.com/1471-230X/11/41/prepub
